# HoxPred: automated classification of Hox proteins using combinations of generalised profiles

**DOI:** 10.1186/1471-2105-8-247

**Published:** 2007-07-12

**Authors:** Morgane Thomas-Chollier, Luc Leyns, Valérie Ledent

**Affiliations:** 1 Belgian EMBnet Node, Université Libre de Bruxelles – CP 257, Bd du Triomphe, B-1050 Brussels, Belgium; 2 Laboratory for Cell Genetics, Vrije Universiteit Brussel, Pleinlaan 2, B-1050 Brussels, Belgium

## Abstract

**Background:**

Correct identification of individual Hox proteins is an essential basis for their study in diverse research fields. Common methods to classify Hox proteins focus on the homeodomain that characterise homeobox transcription factors. Classification is hampered by the high conservation of this short domain. Phylogenetic tree reconstruction is a widely used but time-consuming classification method.

**Results:**

We have developed an automated procedure, HoxPred, that classifies Hox proteins in their groups of homology. The method relies on a discriminant analysis that classifies Hox proteins according to their scores for a combination of protein generalised profiles. 54 generalised profiles dedicated to each Hox homology group were produced *de novo *from a curated dataset of vertebrate Hox proteins. Several classification methods were investigated to select the most accurate discriminant functions. These functions were then incorporated into the HoxPred program.

**Conclusion:**

HoxPred shows a mean accuracy of 97%. Predictions on the recently-sequenced stickleback fish proteome identified 44 Hox proteins, including HoxC1a only found so far in zebrafish. Using the Uniprot databank, we demonstrate that HoxPred can efficiently contribute to large-scale automatic annotation of Hox proteins into their paralogous groups. As orthologous group predictions show a higher risk of misclassification, they should be corroborated by additional supporting evidence. HoxPred is accessible via SOAP and Web interface . Complete datasets, results and source code are available at the same site.

## Background

Hox transcription factors are extensively investigated in diverse fields of molecular and evolutionary biology. This protein family is best known for its crucial role in patterning the anterior-posterior axis of animal embryos [1] and in tetrapod limb development [2]. Hox genes actually belong to the family of homeobox transcription factors characterised by a 60 amino acids region called homeodomain [3].

**Figure 1:**
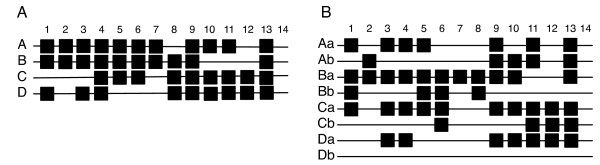
**Hox clusters organisation in vertebrates**. (A) mouse. Hox genes are named following the nomenclature of [32]: The four clusters are assigned to the letters A to D. Hox genes have been numbered 1–14 on each cluster, as gene order is conserved among clusters. (B) zebrafish. The duplicated clusters are specified with a lowercase letter (e.g. Aa, Ab).

Besides, the genomic organisation of Hox genes in clusters is common to most animals. An ancestral Hox gene cluster, supposed to have arisen from tandem duplications in early eukaryotes, has been retained in bilaterians. Hox genes have diverged but the order of each gene along the cluster has been conserved. It is thus possible to assign a given Hox gene by homology to one of the genes along the cluster. Hox genes thus fall into one of the 14 known Paralogous Groups (PG). The ancestral cluster has been duplicated early in the vertebrate lineage (reviewed in [4,5]). Mammals Hox genes are organised in four clusters (Figure 1A) whereas teleost Hox genes (Figure 1B) are generally arranged on 7 clusters, due to an additional duplication specific to teleost fishes [6,7]. Lineage-specific gene loss has subsequently occurred, leading to diverse presence/absence combinations of Hox genes (reviewed in [8]). Cluster duplication have generated paralogues genes that can be grouped in the previously defined PG, for instance HoxA1, HoxB1, HoxC1 and HoxD1 genes belong to PG1. However, each Hox gene of a vertebrate PG is different and will be referred in this study as Orthologous Group (OG) (e.g. mouse and human HoxA1 belong to HoxA1 OG).

The correct identification of individual Hox proteins is an essential basis for their study in evolutionary and developmental research fields. Common methods to classify Hox proteins in their group of homology rely on sequence similarity and phylogenetic analysis. These methods commonly focus on the homeodomain. Classification of Hox proteins is thus hampered by the high conservation of this short domain. Since phylogenetic tree reconstruction is time-consuming, it is not suitable to classify the growing number of Hox sequences. The goal of this work is thus to design an automated procedure that classifies Hox proteins in their groups of homology.

The PROSITE motif databank [9] uses generalised profiles that constitute a scoring system to detect a given motif in new sequences. A generalised profile is a motif descriptor equivalent to a linear hidden Markov model [10]. Although the homeodomain is represented in PROSITE, Hox-specific profiles allowing the precise identification of PG and OG groups have never been defined.

Here, we build Hox generalised profiles dedicated to each PG and OG. These profiles are based on the homeodomain, as the Hox content of an organism is often surveyed by PCR in this region. By using discriminant analysis, we tackle the classification of Hox proteins in their groups of homology, on the basis of their scores for a combination of generalised profiles. Several classification methods are investigated to select the most accurate discriminant functions. These functions are optimised and evaluated on a curated dataset of vertebrate Hox proteins and finally incorporated into the HoxPred program. By applying this program on the Uniprot databank and on two teleost fish proteomes, we demonstrate that HoxPred can efficiently contribute to large-scale annotation of Hox proteins.

## Results

### Evaluation of the Hox-specific generalised profiles

We first evaluated whether Hox-specific profiles can be used to classify Hox sequences in their correct PG and OG. The HOX curated dataset (Table 1) was subdivided to produce 14 PG multiple alignments. A profile was then built out of each alignment. We similarly grouped the HOX set into 40 OG to build the corresponding alignments and profiles.

**Table 1:** Distribution of the 250 sequences composing the HOX dataset

Hox Cluster	PG1	PG2	PG3	PG4	PG5	PG6	PG7	PG8	PG9	PG10	PG11	PG12	PG13	PG14
A	6	8	3	4	4	3	6	-	8	6	8	-	9	1
B	15	6	6	4	6	9	6	3	5	-	-	-	8	-
C	2	-	3	4	5	4	-	3	4	3	1	6	10	-
D	9	-	3	9	1	-	-	4	11	12	6	10	5	1

**Total**	32	14	15	21	16	16	12	10	28	21	15	16	32	2

Classically, the last step of a generalised profile construction is the normalization step that allows the computation of an E-value associated to each profile match [11]. A theoretical cut-off value that separates spurious matches from significant ones is then defined for the calibrated profile. We performed this calibration step for the Hox-specific profiles (Additional files 2). We observed that the theoretical cut-off value is not appropriate to distinguish proteins of a defined PG from other homeoboxes due the high level of residues conservation in the homeodomain.

To evaluate whether each Hox protein can be discriminated by its dedicated profile, we determined the accuracy of each PG and OG profiles in LOO. As an illustration, a PG9-LOO profile is built at each iteration step from the PG9 sequences of the training set, excluding one Hox9 sequence. This PG9-LOO profile is used to score a testing set comprising the excluded Hox9 sequence as positive reference and the non-PG9 sequences of the training set as negative reference. The accuracy of the PG9-LOO profile is calculated for each observed score. The cut-off value of the PG9-LOO profile can thus be defined as the score maximising the accuracy. The PG9 profile mean accuracy is the average of the best accuracies determined at each PG9 iteration.

**Figure 2:**
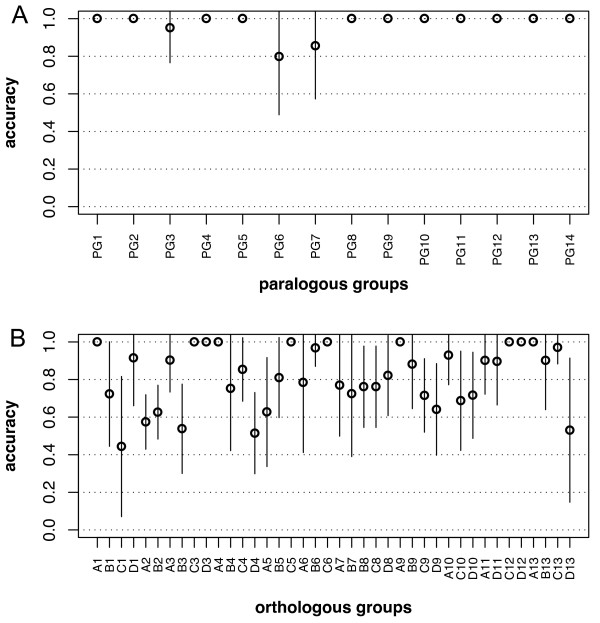
**Accuracy of each Hox group identification with each Hox-specific profile in LOO evaluation**. (A) PG profiles and (B) OG profiles are constructed with BLOSUM100. The average value over all iteration steps is plotted and the dispersion value is the estimated standard deviation

We tested the effect of the substitution matrices used to build the profiles by comparing the results obtained with 3 BLOSUM matrices. Figure 2 summarises the results for the evaluation of all Hox-specific profiles built with BLOSUM100. The accuracy for PG profiles (Figure 2A) is very high except for PG3, PG6 and PG7 where the mean value is nevertheless always above 80%. Figure 2B shows that the mean accuracy for OG profiles is very variable, even between OG belonging to the same PG. Most profiles have mean accuracy values lower than 80% and the dispersion values are quite high. Results obtained with BLOSUM65 and BLOSUM80 always showed lower or equal accuracy values (data not shown).

It is thus possible to distinguish the PG of vertebrate Hox proteins with a reasonable confidence by using PG-specific profiles. Differentiating the OG on the basis of a single profile is, however, not suitable since the proportions of false predictions are excessively high for some groups. We therefore evaluated whether combining information of several profiles could improve the discrimination between highly similar sequences. As discriminant analysis proved efficient to combine information of position-specific scoring matrices (PSSM) to classify genes on the basis of putative regulatory elements [12], we applied this method on the Hox classification problem. The major novelties of this approach are the use of generalised profiles technology instead of PSSM and the application of discriminant analysis to classify protein sequences.

### Discriminant analysis based on generalised profile scores

We applied discriminant analysis with a view to predict Hox PG and OG, on the basis of multiple scores obtained with our previously produced Hox-specific profiles. As profile thresholds are not taken into account in the discriminant analysis, we used Hox-specific profiles without defining cut-off values. Each evaluation was performed in LOO within a forward stepwise variable selection to prevent the risk of over-fitting and to define the optimal subset of profiles. We compared the results obtained with 4 classification methods (*2-groups*, *all-groups*, *anterior*/*posterior *and *PG-groups*) to select the most accurate method to predict each Hox group. To conduct the discriminant analyses, the prior probabilities were set to a very high value (*>*99%) for the control (CTL) group and to 0,0001 for each Hox class.

#### Variable selection

**Figure 3:**
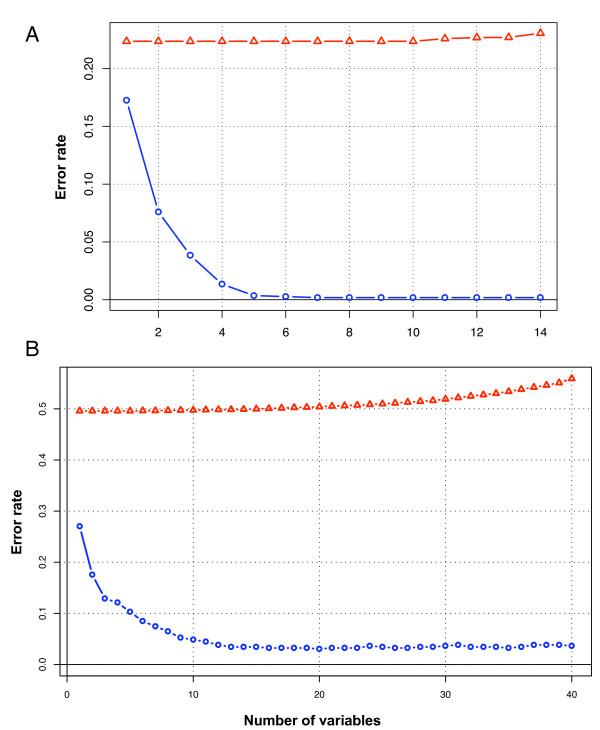
**Variables selection for the all-group classification **. Error rates obtained with linear discriminant analysis approach in LOO, on real and permuted data, as a function of the number of variables selected by the forward stepwise procedure. (A) *all-groups *classification for PG profiles (B) *all-groups *classification of OG profiles. Symbols : circle, real data; triangle, permuted data

Figure 3 displays results of the variable selection with LDA method on the real or permuted dataset. At each iteration of this analysis, a prediction function was built with an increasing number of variables and the error rate was calculated. The error rates are plotted as a function of the number of selected variables.

For Hox PG prediction, we performed a training with the *all-groups *classification (Table 2, Figure 3A). The error rate decreases as variables are incorporated and reach its lowest value (0.17%) for 5–14 variables. The error rates on the permuted set are stable at 22% of errors, which corresponds to a random classification in the CTL class that exhibits the highest prior probability.

**Table 2:** List of training datasets and profiles for the four classification methods

classification method	training dataset composition		variables
	Hox sequences	CTL sequences	
**all-groups**			
(PG)	HOX	RANDOM + HOMEO	all 14 PG profiles
(OG)	HOX	RANDOM	all 40 0G profiles

**2-groups**	HOX	RANDOM	all 40 0G profiles
	(2 classes: thisOG or notThisOG)		

**anterior/posterior**			
anterior	HOX:PG1–8 only	RANDOM	OG profiles of PG1–8
posterior	HOX:PG9–13 only	RANDOM	OG profiles of PG9–13

**PG-groups**	HOX: OG of a single PG	RANDOM	OG profiles of a single PG

We applied the 4 classification methods for the training of Hox OG prediction. For each method, a variable selection step is performed to define the optimal subset of ordered variables, as illustrated for the *all-groups *classification in figure 3B. The error rate first decreases rapidly until 13 variables are incorporated, and then slightly oscillate around 3.5% of errors. The optimal discrimination is obtained with 3% of errors (20 variables) for this type of classification. A random classification (permutated dataset) returns error rates of 50% when all sequences of the training set are predicted as CTL. It is interesting to observe the increase of the error rate when more than 20 variables are incorporated. This effect strongly suggests a situation of over-fitting since training is performed with more variables (20–40 profiles) than elements in each class (less than 20 sequences, Table 1).

#### Selection of optimal classification methods

With *all-groups *method, the optimal linear discriminant function using all 14 variables (Table 5) classifies in a very stringent way Hox sequences in their correct PG. The confusion table (Table 3) summarizes this classification in PG, trained in LOO with the *all-groups *method.

**Table 3:** Confusion table of HOX and CTL training sets for PG predictions with all-groups method

	training														
pred.	CTL	PG1	PG10	PG11	PG12	PG13	PG14	PG2	PG3	PG4	PG5	PG6	PG7	PG8	PG9
CTL	**866**	0	0	0	0	0	0	0	0	0	0	0	0	0	0
PG1	**1**	**32**	0	0	0	0	0	0	0	0	0	0	0	0	0
PG10	0	0	**21**	0	0	0	0	0	0	0	0	0	0	0	0
PG11	0	0	0	**15**	0	0	0	0	0	0	0	0	0	0	0
PG12	0	0	0	0	**16**	0	0	0	0	0	0	0	0	0	0
PG13	0	0	0	0	0	**32**	0	0	0	0	0	0	0	0	0
PG14	0	0	0	0	0	0	**2**	0	0	0	0	0	0	0	0
PG2	0	0	0	0	0	0	0	**14**	0	0	0	0	0	0	0
PG3	0	0	0	0	0	0	0	0	**15**	0	0	0	0	0	0
PG4	0	0	0	0	0	0	0	0	0	**21**	0	0	0	0	0
PG5	0	0	0	0	0	0	0	0	0	0	**16**	0	0	0	0
PG6	0	0	0	0	0	0	0	0	0	0	0	**16**	0	0	0
PG7	**1**	0	0	0	0	0	0	0	0	0	0	0	**12**	0	0
PG8	0	0	0	0	0	0	0	0	0	0	0	0	0	**10**	0
PG9	0	0	0	0	0	0	0	0	0	0	0	0	0	0	**28**

The optimal linear discriminant function was defined by the variable selection step. Error rate: 0.17%

Two CTL sequences corresponding to the homeobox HM1_CHICK and HMSA_SALSA, were identified respectively as PG1 and PG7 in our analysis. By querying HM1_CHICK with BLASTP [13] against the chick proteome at Ensembl, HM1_CHICK matches an Ensembl gene prediction located near the chick HoxD cluster and highly similar to HoxD1 genes of mammals. Even though HMSA_SALSA is not annotated as Hox, this salmon sequence has been previously considered as HoxA7 [14]. It is thus reasonable to consider these two sequences as true Hox genes correctly classified by the discriminant function but misannotated in the original database.

**Figure 4:**
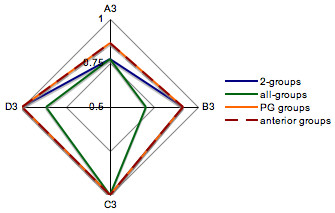
**Radar plot of OG prediction accuracies with the 4 classification methods, within PG3**. Each classification method is represented as a polygon. As PG3 contains 4 OG, the polygons are quadrilateral. The most performant method is represented as the polygon having the larger surface.

For OG predictions, we tested the 4 classification methods and selected the method that best predicts all OG within a given PG. In order to compare the performance of the 4 methods, we calculated the accuracy of each OG prediction with each method in LOO. Within each PG, accuracies of OG predictions were displayed on a radar plot so that each classification method is represented as a polygon, as illustrated for PG3 in Figure 4. The most effective method is thus represented as the polygon having the larger surface. Table 4 summarizes the surface of each polygon for the 13 PG.

**Table 4:** Comparison of the 4 classification methods performance for OG predictions among each PG.

PG	2-groups	PG-groups	anterior/posterior	all-groups
PG1	2.00	2.00	2.00	2.00
PG2	0.73	0.60	0.62	0.62
PG3	1.70	1.78	1.78	1.40
PG4	1.70	1.65	1.60	1.82
PG5	0.43	0.43	0.43	0.38
PG6	0.43	0.43	0.43	0.43
PG7	1.00	0.85	0.85	0.85
PG8	0.40	0.43	0.43	0.36
PG9	1.89	1.85	1.63	1.63
PG10	0.40	0.43	0.43	0.39
PG11	1.00	1.00	1.00	1.00
PG12	1.00	1.00	1.00	1.00
PG13	1.89	1.85	1.85	1.85

Each value is the surface of the polygon that represents one classification method on a radar plot. The polygon is quadrilateral for PG containing 4 OG and triangular for PG containing 3 OG. For PG containing 2 OG, the value is the product of the 2 OG accuracies, for a given classification method.

**Table 5:** Optimal classification methods with their corresponding discriminant functions

PG	classification	nb variable	variable name and order
*PG classification*			
-	all-groups	14	PG7 PG9 PG1 PG2 PG6 PG13 PG11 PG10 PG3 PG12 PG14 PG4 PG8 PG5
*OG classification*			
PG1	anterior	18	C6 B2 D4 C3 B6 D1 B7 A7 C4 B4 A5 C1 A2 A4 C8 D8 B5 A6
PG2	2-groups	A2 : 10	A2 A3 B2 A5 A4 A6 A7 B4 B6 C4
		B2 : 8	B2 A2 A5 A6 A7 B7 B8 C6
PG3	anterior	18	*same as PG1*
PG4	all-groups	20	C6 D9 D13 D1 C13 C4 B3 A11 B1 A7 A9 C3 B4 C1 A6 A1 A4 D11 C5 B9
PG5	anterior	18	*same as PG1*
PG6	anterior	18	*same as PG1*
PG7	2-groups	A7 : 19	A7 B6 A5 B5 A1 A2 A3 A6 B1 B2 A4 B3 B4 C1 C3 C4 D1 C5 D4
		B7 : 24	B7 A7 A1 B6 D8 A4 A2 A3 A5 B1 B2 B3 B5 A6 B8 C3 D1 C1 C4 C5 D3 B4 C8 D4
PG8	anterior	18	*same as PG1*
PG9	PG-groups	4	A9 B9 C9 D9
PG10	posterior	14	B9 D13 A9 A13 A10 D9 C10 C9 C13 A11 B13 C12 D11 D12
PG11	posterior	14	*same as PG10*
PG12	posterior	14	*same as PG10*
PG13	posterior	14	*same as PG10*

The order and number of variables defined in the variable selection step are listed.

Contrary to PG predictions, no single classification method is adequate to accurately predict all OG. Table 5 summarizes the selected optimal methods to predict OG within each PG. Among several suitable functions within a PG, the *anterior*/*posterior *classification method was favoured to ensure a restricted number of functions to manipulate. For Hox sequences of the posterior groups (9–13), the OG sequences of PG10–13 are predicted with a higher confidence by *posterior *method. Although PG9 belongs to the posterior group, its optimal method is *PG-groups*. For anterior groups (1–8), *anterior *classification is the most accurate to predict OG sequences of PG1, PG3, PG5, PG6 and PG8. Classification of sequences in OG belonging to PG2 and PG7, however, shows better results with the *2-groups *method. Last, PG4 is the only PG exhibiting greater accuracy with the *all-groups *classification.

#### Single-profile technique versus discriminant analysis

**Figure 5:**
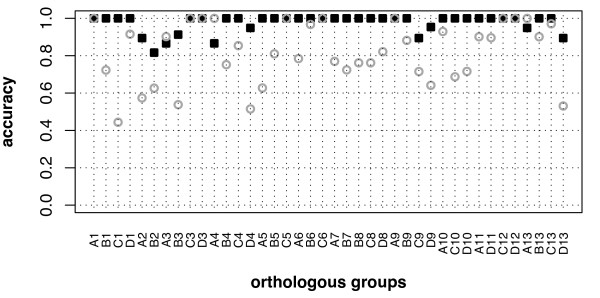
**Comparison of OG predictions accuracy with single-profile method and discriminant analysis**. Accuracy of OG identification with single-profile method and discriminant analysis conducted with optimal classification methods and their corresponding discriminant functions, in LOO. For single-profile method, the average value is plotted and the dispersion value is omitted for clarity (see Figure 2B for dispersion plot) Symbols : circle, single-profile; square, discriminant analysis

To determine whether combining profiles yields more accurate PG and OG predictions, we compared the accuracy in LOO of both single-profile technique and optimal discriminant functions. As discriminant analysis classifies all PG with 100% accuracy, we favoured this method over single-profile technique. Similarly, the mean accuracy for OG predictions is significantly higher for the discriminant analysis (97%) than for the single profile technique (81%). Figure 5 plots the accuracy obtained with single-profile technique and discriminant analysis as a function of the 40 OG. With the discriminant functions, all OG are predicted with accuracy higher than 80%. We noted only three OG (HoxA3, HoxA4 and HoxA13) where single-profile method is slightly more accurate. The few OG misclassifications observed are nevertheless confined to the correct PG. PG2 predictions appear to be the less accurate, mainly because mouse HoxA2 and HoxB2 homeodomain sequences are identical. In PG3, the amphibian B3_PLEWA is misclassified as A3 with a posterior probability of 0.5 while the proper identification as B3 has a probability of 0.46. Likewise for PG9, the divergent fish sequence D9a_ORYLA is predicted as C9 with posterior probabilities of 0.5 and 0.49 for D9, the correct OG for this protein. Conversely, misclassifications with a posterior probability of 1 are observed for the A4_MOUSE and shark D13_HETFR sequences that are predicted as D4 and A13, respectively. A4_MOUSE actually differs from D4_MOUSE by only one residue and its vector of scores, used to train discriminant analysis, is much more similar to D4 group than A4 group.

In summary, the discriminant analysis provides a very stringent function that classifies sequences in PG on the basis of all PG profiles. A set of optimal functions predicts OG with a high accuracy, on the basis of carefully defined subsets of OG profiles.

#### HoxPred

The optimal discriminant functions were incorporated in a program called HoxPred. To fully exploit the potential of this program, the sequences submitted should contain 60aa centered on the homeodomain. First, the submitted sequence is scored by the 14 PG profiles. These scores then serve as input for the PG discriminant function. Unless the sequence is predicted as CTL, the program adjusts the OG analysis to the optimal classification method for the predicted PG. The output is a XML-formatted file that reports the PG and OG predictions and their associated posterior probabilities. This program is available as a Simple Object Access Protocol (SOAP) server.

### Application of HoxPred to UniProt homeobox proteins

To test HoxPred on a wide range of proteins and detect unsuspected false positive predictions, we applied it to the UniProt databank. As UniprotKB (Trembl 34.1 and Swissprot 50.9) contain more than 3 millions sequences, we first extracted the homeobox sequences to reduce the number of sequences to be analyzed with HoxPred. UniprotKB was thus first filtered with the homeobox Prosite Profile PS50071 and the resulting 7155 Trembl sequences and 1131 Swissprot sequences were then submitted to HoxPred (Additional file 3). Except the misannotated HM1_CHICK and HMSA_SALSA already mentioned above, no additional false positive was detected since non-Hox homeobox are correctly classified as CTL. Some misclassifications are nevertheless noticeable, such as PG2 predictions or sequences identical to A4_MOUSE that are all predicted as D4. We especially noticed the HXD3_CHICK prediction as PG3/A3. While PG prediction is correct, the OG classification seems erroneous. Multiple alignment of full-length PG3 sequences reveal that HXD3_CHICK is more similar to A3 than D3, which argues in favour of a misannotation of HXD3_CHICK.

The Uniprot databank comprises many short fragments (*<*60 residues) produced by PCR surveys. As profile scoring-system is length-dependent, input protein fragments for HoxPred should be at least as long as the profile (60 residues) and span the homeodomain. Despite this limitation, we observed that 98% of the complete set of 64 amphibian PCR fragments (39 residues) [15] is correctly classified as regards to PG (Additional file 4). PG predictions seem thus quite robust to short fragments. Also, 69% of these sequences were correctly classified in OG.

Interesting results are obtained with non-vertebrate sequences (Additional file 3). Indeed, positive predictions encompass sequences of more distant deuterostomes and protostomes. For non-vertebrate organisms, only PG prediction is meaningful as Hox genes are organised in a single cluster. Based on phylogenetic reconstructions, bilaterian Hox genes can be classified into four groups: Anterior (PG1–2), Group 3 (PG3), Central (PG4–8) and Posterior (PG9–14), according to their position in the cluster and site of expression along the anteroposterior axis [16]. Preliminary analyses show that HoxPred predictions are consistent with the commonly accepted Hox homology relationships between vertebrate and more distant organisms [17] into these 4 groups.

### Fishing Hox out of the Gasterosteus aculeatus proteome

In order to assess the potential of HoxPred for genome-scale analyses, we applied HoxPred to the proteomes of two closely related fishes: *Oryzia latipes *(medaka) and *Gasterosteus aculeatus *(stickleback). Proteomes were retrieved from Ensembl v40, filtered with the homeobox Prosite Profile PS50071 and then submitted to HoxPred. HoxPred predictions were then sorted by chromosome to highlight the Hox clusters organisation.

For the medaka, HoxPred predictions are located on 7 chromosomes (Additional file 5, Figure 6A), which corroborates the 7 clusters organisation reported in [8]. Among the 48 Hox proteins reported in [8], 5 are absent from the Ensembl gene predictions (HoxA7a, HoxC6a, HoxB7a, HoxB8a and HoxD11b) likely due to gaps in the genome assembly. HoxPred correctly predicts all 43 remaining Hox proteins with respect to PG and only 2 OG predictions were erroneous. Indeed, the HoxD11 prediction located on the HoxCa cluster and the HoxA2 prediction located on the HoxBa cluster are misclassified and respectively correspond to HoxCa11 and HoxB2a (Additional file 5).

**Figure 6:**
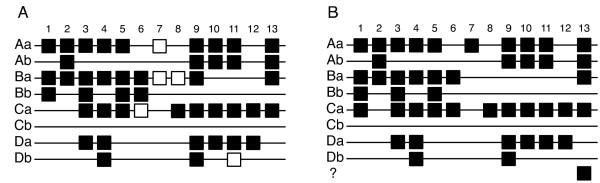
**Putative Hox genes organisation for medaka and stickleback**. Hox genes organisation for medaka (A) and stickleback (B), deduced from HoxPred predictions. Symbols: black square, identified gene ; white square, hypothetical gene reported in [8], not present in Ensembl.

In stickleback, HoxPred returns 45 predictions, which encompass the 10 stickleback Hox genes previously identified in [18]. Predictions are consistent with a 7 clusters organisation, except one Hox lying external to the 7 clusters (Additional file 5, Figure 6B). The homeodomain sequence of this additional protein, predicted as PG13/B13, is identical to the HoxB13a homeodomain. For the 44 remaining Hox proteins, HoxPred predictions were validated by comparing them with the order of the corresponding genes on the genome assembly. All Hox PG are correctly predicted. For OG predictions, HoxB2a and HoxC11a are respectively misclassified as A2 and D11, as observed in medaka. Besides, genome assembly allows us to locate a PG1/B1 prediction on the HoxCa cluster, at the position of a potential HoxC1a. Phylogenetic reconstructions (Additional file 6) have confirmed that this protein is a HoxC1a.

Actually, Hox organisation in medaka and stickleback are highly similar with 40 OG in common. Among the five Hox genes not found in the current medaka assembly but reported in [8], HoxA7a and HoxC6a genes are present in stickleback, but not HoxB7a, HoxB8a and HoxD11b. Gaps in the stickleback genome assembly or fused gene predictions in Ensembl may explain the absence of HoxPred predictions for these OG.

## Discussion

Classifying Hox proteins in their groups of homology is a time-consuming and complex task due to the highly conserved homeodomain. The goal of this project was to develop an automated classification program that is appropriate to classify the growing number of Hox sequences. This program, HoxPred, relies on a method that combines Hox-specific protein generalised profiles and discriminant analysis to distinguish Hox PG and OG, despite the high similarity between their homeodomain sequences. Applied on a curated dataset of vertebrate Hox proteins, HoxPred predicts all PG correctly and shows a mean accuracy of 97% for OG predictions.

We first have shown that the single-profile technique yields relatively good results for PG but global accuracy is not sufficient for OG predictions. Generalised profiles can be manually edited with a view to increase their discriminative power by modifying scores of diagnostic residues at specific positions of the motif [11]. Defining diagnostic residues specific to each group of homology is problematic in the case of Hox multigenic family [15]. As more divergent Hox sequences become available, diagnostic residues could be altered and it would require to individually re-edit up to 54 profiles manually. Consequently, we opted for a global and automated approach, which combines profiles and discriminant analysis. Discriminant analysis evaluation revealed a significant increase of accuracy for both PG and OG predictions compared to single-profile technique. In addition, discriminant functions avoid the need for profile thresholds. The combination of profiles thus provides better information to distinguish both Hox PG and OG. Restricting the number of profiles is nevertheless essential to avoid risk of over-fitting.

As the evaluation was performed in LOO, the usage of a non-redundant set of training sequences was mandatory. Additional and divergent sequences would be valuable to allow HoxPred to predict OG HoxA14, HoxD14, HoxC11, HoxD10 and HoxB10. Indeed, these groups are represented by only one non-redundant sequence that cannot be analysed in LOO. We tested both LDA and QDA methods and observed that LDA gave better results than QDA. As previously suggested [12], this situation may be explained by the small size of the training set. Moreover, we tested more sophisticated SVM methods and obtained classifications of lower accuracy, especially for OG (not shown). To avoid misclassifications, we deliberately chose a very high prior probability (99%) for the CTL groups. Permutation tests clearly showed that predictions are randomly classified in the CTL group rather than in another group. Although this choice of prior probability could have had a cost in terms of sensitivity, we did not observe false negatives due to misclassifications of OG sequences in the CTL group. Rather, we observed that misclassifications are restricted to the same PG. Though often informative, posterior probabilities returned by the discriminant function should be interpreted with care since we observed some misclassifications with a probability of 1.

An automated homeobox classification method, P-Gnome, had previously been proposed [19]. It relies on the determination of phylogenetically characteristic residues of Hox proteins in a guide tree. In order to compare the performance of HoxPred and P-Gnome on Hox proteins classification, P-Gnome was applied to our HOX dataset. With P-Gnome, only 27.2% of the HOX dataset is correctly classified as regards to PG and 11.6% as regards to OG (versus 100% and 96% respectively for HoxPred in LOO). As the combination of characteristic residues employed by P-Gnome does not take into account the variability brought by newly sequenced Hox proteins, we first updated its training dataset and re-evaluate its performance. When trained with a bayesian phylogenetic tree constructed on our HOX dataset, P-Gnome correctly classifies 74.4% the HOX dataset in PG and 36% in OG. The quality of the predictions was lower with a parsimony phylogenetic tree. HoxPred therefore performs significantly better than this alternative automated method.

We demonstrated that HoxPred is appropriate to decipher Hox proteins from whole genomes by applying it on two telost fishes. Predictions were largely correct even though teleost Hox sequences are known to be divergent consequently to additional duplication of their Hox clusters. We characterized the Hox content of the recently sequenced stickleback genome, based on Ensembl automatic annotation. No stickleback sequences were used to construct the profiles for HoxPred. We identified 44 proteins belonging to 7 clusters and suggest that stickleback comprises a HoxC1a gene, only found so far in zebrafish. A PG13/B13 prediction outside of the clusters raises questions about the origin of the corresponding gene. As its homeodomain is identical to HoxB13a, it could result from a lineage-specific duplication of HoxB13a, further displaced. Since the Ensembl gene prediction spans over a gap in the assembly, more accurate predictions could be achieved as the genome sequence is completed.

By applying HoxPred to Swissprot, we reassigned 2 proteins annotated as homeobox (HM1_CHICK and HMSA_SALSA) to true Hox. HoxPred also detected a misannotation of HXD3_CHICK sequence that actually belongs to HoxA3 group.

## Conclusion

HoxPred correctly discriminates Hox sequences from non-Hox homeoboxes, including the closely related paraHox proteins. This study indicates that HoxPred can efficiently contribute to a better annotation of Hox in vertebrates. HoxPred is particularly appropriate for automatic classification of Hox proteins into their paralogous groups. As orthologous group predictions show a higher risk of misclassification, they should be corroborated by additional supporting evidence. The computational time for HoxPred (5 min on a PowerMac G5 2.5 GHz for 250 sequences) and its availability as a SOAP server allow its integration in a workflow for large-scale Hox annotation.

The Hox content of many organisms is often analysed by PCR surveys that produce very short sequence fragments. We showed that HoxPred could help identifying PG in PCR surveys. Besides, the application of HoxPred on a wide range of organisms revealed that non-vertebrate Hox proteins also matched vertebrate Hox-specific profiles. Classification of Hox proteins is particularly challenging for invertebrates and is being actively debated for evo-devo model species such as those belonging to cnidarians [20]. Cnidarian sequences have been described as highly divergent Hox sequences, difficult to relate to the different bilaterian Hox groups. The order of the genes on Hox clusters is valuable information for the classification in groups of homology. Classification is thus hampered in species where Hox gene clusters are desintegrated, such as in urochordates [21]. The application field of HoxPred could be extended to bilaterians and more distant eumetazoa, and could bring interesting insights for taxa where phylogeny-based Hox classifications are indecisive.

## Methods

### Hox homeodomains training set

Vertebrate Hox protein sequences were collected from phylogenetic studies in the literature (Additional files 1) and the SwissProt Hox list release 49.7 [22,23] at [24]. Additional mammal sequences were retrieved from Ensembl v40 with BioMart [25]. The 853 sequences of this initial dataset were grouped by OG and aligned with ClustalX [26]. For each OG sequence, the homeodomain was extracted. This homeodomain dataset was manually curated by excluding partial, misannotated and lower-quality sequences. To avoid over-representation of subfamilies, we removed identical homeodomains among each OG. The resulting dataset (HOX) comprises 250 non-redundant Hox homeodomains. A group of 250 random sequences (RANDOM) was generated by RSAT [27] to display the same lengths as the 250 full-length sequences of the HOX dataset. The third group (HOMEOBOX) comprises 618 vertebrate proteins from Swissprot 50.9, which match the homeobox motif Prosite:PS50071 [28] but are not annotated as Hox.

### Generalised profiles

#### Profiles construction

Generalised profiles [10] were constructed with the PFTOOLS package, as described in [11]. Two types of profiles, hereafter referred to as 'PG profiles' and 'OG profiles', were developed. Sequences of HOX dataset were first grouped into PG and OG and aligned with ClustalX. Each sequence of the 14 PG and 40 OG alignments was weighted by using the pfw program of the PFTOOLS package (number of shuffles per sequence = 2000). The weighted alignments were converted into profiles with the program pfmake in semi-global alignment mode. We tested the BLOSUM65, BLOSUM80 and BLOSUM100 [29] substitution matrices to construct the profiles. The program pfsearch performed the alignments of the profiles against protein sequences. This program was set to return for each sequence a unique score assigned to the optimal alignment.

#### Leave-one-out evaluation

Given the small number of non-redundant Hox homeodomains (often less than 5 by OG) (Table 1), we could not consider dividing them into smaller subsets. To evaluate the accuracy of the profiles, we thus applied a leave-one-out (LOO) cross-validation procedure [30]. Each multiple alignment, excluding one sequence, serves as training set to construct the profile. This profile is then searched against the testing set that comprises the excluded sequence, the remainders of the HOX dataset and the RANDOM dataset. The procedure is repeated for all sequences of the multiple alignment. The geometric accuracy is the statistic used to evaluate the performance of the profiles. Accuracy = Sn.PPV
 MathType@MTEF@5@5@+=feaafiart1ev1aaatCvAUfKttLearuWrP9MDH5MBPbIqV92AaeXatLxBI9gBaebbnrfifHhDYfgasaacH8akY=wiFfYdH8Gipec8Eeeu0xXdbba9frFj0=OqFfea0dXdd9vqai=hGuQ8kuc9pgc9s8qqaq=dirpe0xb9q8qiLsFr0=vr0=vr0dc8meaabaqaciaacaGaaeqabaqabeGadaaakeaadaGcaaqaaiabdofatjabd6gaUjabc6caUiabdcfaqjabdcfaqjabdAfawbWcbeaaaaa@33C6@, where the sensitivity Sn = TP/(TP + FN) and the positive predictive value PPV = TP/(TP + FP), with TP, TN, FP and FN referring to the number of True Positives, True Negatives, False Positives and False Negatives, respectively. Accuracy is averaged over each iteration step.

### Discriminant analysis

We tested both the Linear Discriminant Analysis (LDA) and Quadratic Discriminant Analysis (QDA) to classify the sequences in Hox PG and OG according to their scores for the different Hox PG and OG generalised profiles. Discriminant analysis was performed with the statistical package R [31]. We optimized the discriminant functions and evaluated the predictive performance of LDA and QDA as described in [12]. Briefly, a variable selection step was performed within a forward stepwise procedure so as to select the subset of ordered variables that are the most discriminating. The predictive power of discriminant functions was evaluated with a LOO procedure. The labels of the training dataset were permuted to evaluate the rate of correct predictions that the discriminant functions returns by chance. For each discriminant function, 100 independent permutation tests were performed.

### Four classification methods

We defined four classification methods and systematically compared the results obtained with these methods to select the most accurate for each Hox group. A classification method is specified by the score matrix used to train the discriminant analysis. Each protein sequence of the training dataset is represented in the matrix by a profile score vector, which length equals the number of profiles to be used as variables in the discriminant analysis. Table 2 summarises the training dataset and profiles that were used to constitute the score matrix of each classification method.

First, the *all-groups *classification method consists of a single prediction function that directly assigns an element to a Hox class (PG or OG) or to the control (CTL) class. The second method aims at classifying the elements in 2 groups: the correct group versus the not correct group (e.g. HoxA9 versus notA9). This *2-groups *method produces one prediction function for each Hox group. The third classification method restricts the analysis either to sequences of PG1–8 referred to as *anterior *or PG9–13 referred to as *posterior*. Last, the *PG-groups *method restricts the training set and profiles to a specific PG. Even if the number of profiles is reduced in the two latter methods, we performed the variable selection step to limit the risk of over-fitting.

## Availability and requirements

Project name: HoxPred

Project home page: 

Operating system(s): Unix

Programming language: Java and R

Other requirements: Tomcat 5.5 or higher

License: GNU GPL

Any restrictions to use by non-academics: None

## Authors' contributions

MT-C conceived the study. VL performed the phylogenetic analyses. VL, MT-C and LL participated in the coordination of the study. MT-C and VL drafted the manuscript and all the authors participated in the editing of the manuscript. All the authors read and approved the final manuscript.

## Supplementary Material

Additional File 1 Accession numbers of the Hox sequences used in this study Click here for file

Additional File 2 Distribution of scores for the alignment of PG9 profile against Swissprot and randomized protein sequences Click here for file

Additional File 3 Application of HoxPred to UniProt homeobox proteins Click here for file

Additional File 4 Application of HoxPred to 64 amphibian PCR fragments Click here for file

Additional File 5 Application of HoxPred to medaka and stickleback homeobox proteins Click here for file

Additional File 6 Phylogenetic tree of PG1 in selected teleost fishes Click here for file

## References

[B1] McGinnis W, Krumlauf R (1992). Homeobox genes and axial patterning. Cell.

[B2] Nelson CE, Morgan BA, Burke AC, Laufer E, DiMambro E, Murtaugh LC, Gonzales E, Tessarollo L, Parada LF, Tabin C (1996). Analysis of Hox gene expression in the chick limb bud. Development.

[B3] Gehring WJ, Muller M, Affolter M, Percival-Smith A, Billeter M, Qian YQ, Otting G, Wuthrich K (1990). The structure of the homeodomain and its functional implications. Trends Genet.

[B4] Finnerty JR, Martindale MQ (1998). The evolution of the Hox cluster: insights from outgroups. Curr Opin Genet Dev.

[B5] Prince V (2002). The Hox Paradox: More complex(es) than imagined. Dev Biol.

[B6] Amores A, Force A, Yan YL, Joly L, Amemiya C, Fritz A, Ho RK, Langeland J, Prince V, Wang YL, Westerfield M, Ekker M, Postlethwait JH (1998). Zebrafish hox clusters and vertebrate genome evolution. Science.

[B7] Crow KD, Stadler PF, Lynch VJ, Amemiya C, Wagner GP (2006). The "fish-specific" Hox cluster duplication is coincident with the origin of teleosts. Mol Biol Evol.

[B8] Hoegg S, Meyer A (2005). Hox clusters as models for vertebrate genome evolution. Trends Genet.

[B9] Hulo N, Bairoch A, Bulliard V, Cerutti L, De Castro E, Langendijk-Genevaux PS, Pagni M, Sigrist CJA (2006). The PROSITE database. Nucleic Acids Res.

[B10] Bucher P, Karplus K, Moeri N, Hofmann K (1996). A flexible motif search technique based on generalized profiles. Comput Chem.

[B11] Sigrist CJA, Cerutti L, Hulo N, Gattiker A, Falquet L, Pagni M, Bairoch A, Bucher P (2002). PROSITE: a documented database using patterns and profiles as motif descriptors. Brief Bioinform.

[B12] Gonze D, Pinloche S, Gascuel O, van Helden J (2005). Discrimination of yeast genes involved in methionine and phosphate metabolism on the basis of upstream motifs. Bioinformatics.

[B13] Altschul SF, Madden TL, Schaffer AA, Zhang J, Zhang Z, Miller W, Lipman DJ (1997). Gapped BLAST and PSI-BLAST: a new generation of protein database search programs. Nucleic Acids Res.

[B14] Moghadam HK, Ferguson MM, Danzmann RG (2005). Evidence for Hox gene duplication in rainbow trout (Oncorhynchus mykiss): a tetraploid model species. J Mol Evol.

[B15] Mannaert A, Roelants K, Bossuyt F, Leyns L (2006). A PCR survey for posterior Hox genes in amphibians. Mol Phylogenet Evol.

[B16] Garcia-Fernandez J (2005). The genesis and evolution of homeobox gene clusters. Nat Rev Genet.

[B17] Balavoine G, de Rosa R, Adoutte A (2002). Hox clusters and bilaterian phylogeny. Mol Phylogenet Evol.

[B18] Ahn DG, Gibson G (1999). Expression patterns of threespine stickleback hox genes and insights into the evolution of the vertebrate body axis. Dev Genes Evol.

[B19] Sarkar IN, Thornton JW, Planet PJ, Figurski DH, Schierwater B, DeSalle R (2002). An automated phylogenetic key for classifying homeoboxes. Mol Phylogenet Evol.

[B20] Ryan JF, Mazza ME, Pang K, Matus DQ, Baxevanis AD, Martindale MQ, Finnerty JR (2007). Pre-Bilaterian Origins of the Hox Cluster and the Hox Code: Evidence from the Sea Anemone, Nematostella vectensis. PLoS ONE.

[B21] Lemons D, McGinnis W (2006). Genomic evolution of Hox gene clusters. Science.

[B22] Bairoch A, Apweiler R (1997). The SWISS-PROT protein sequence database: its relevance to human molecular medical research. J Mol Med.

[B23] Wu CH, Apweiler R, Bairoch A, Natale DA, Barker WC, Boeckmann B, Ferro S, Gasteiger E, Huang H, Lopez R, Magrane M, Martin MJ, Mazumder R, O'Donovan C, Redaschi N, Suzek B (2006). The Universal Protein Resource (UniProt): an expanding universe of protein information. Nucleic Acids Res.

[B24] SwissProt Hox list. http://www.expasy.org/cgi-bin/lists?hoxlist.txt.

[B25] Birney E, Andrews D, Caccamo M, Chen Y, Clarke L, Coates G, Cox T, Cunningham F, Curwen V, Cutts T, Down T, Durbin R, Fernandez-Suarez XM, Flicek P, Graf S, Hammond M, Herrero J, Howe K, Iyer V, Jekosch K, Kahari A, Kasprzyk A, Keefe D, Kokocinski F, Kulesha E, London D, Longden I, Melsopp C, Meidl P, Overduin B, Parker A, Proctor G, Prlic A, Rae M, Rios D, Redmond S, Schuster M, Sealy I, Searle S, Severin J, Slater G, Smedley D, Smith J, Stabenau A, Stalker J, Trevanion S, Ureta-Vidal A, Vogel J, White S, Woodwark C, Hubbard TJP (2006). Ensembl 2006. Nucleic Acids Res.

[B26] Thompson JD, Gibson TJ, Plewniak F, Jeanmougin F, Higgins DG (1997). The CLUSTAL_X windows interface: flexible strategies for multiple sequence alignment aided by quality analysis tools. Nucleic Acids Res.

[B27] van Helden J (2003). Regulatory sequence analysis tools. Nucleic Acids Res.

[B28] Prosite database. http://www.expasy.org/prosite.

[B29] Henikoff S, Henikoff JG (1992). Amino acid substitution matrices from protein blocks. Proc Natl Acad Sci USA.

[B30] Huberty CJ (1994). Applied Discriminant Analysis.

[B31] Statistical package R. http://www.r-project.org.

[B32] Scott MP (1993). A rational nomenclature for vertebrate homeobox (HOX) genes. Nucleic Acids Res.

