# Supplementary material for: HoxPred: automated classification of Hox proteins using combinations of generalised profiles
Source: BMC Bioinformatics. 2007 Jul 12;8:247. doi: 10.1186/1471-2105-8-247 (PMC1965487; doi:10.1186/1471-2105-8-247)
Supplement: Additional File 2 — Distribution of scores for the alignment of PG9 profile against Swissprot and randomized protein sequences [file 1471-2105-8-247-S2.pdf]

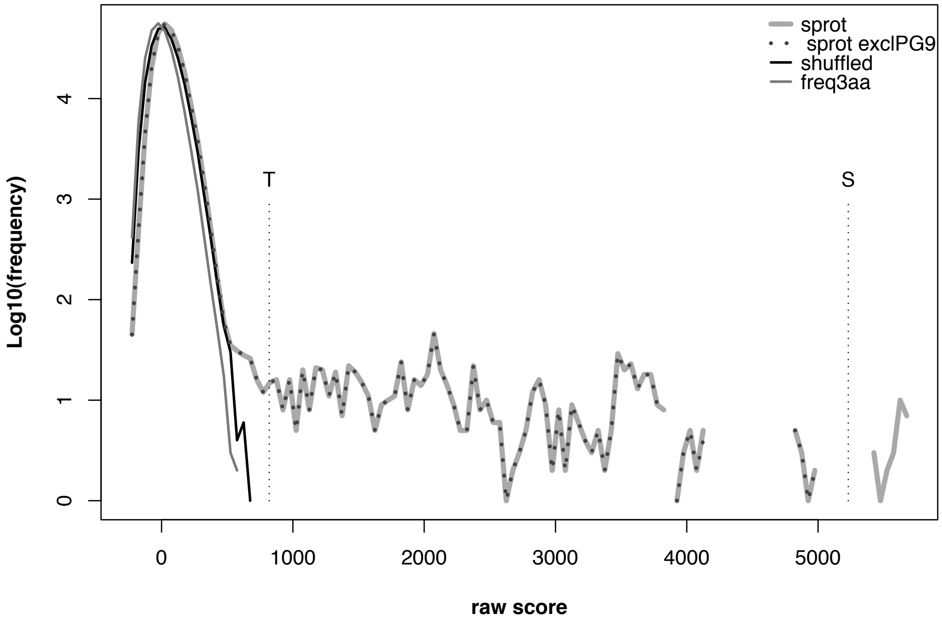

Figure 1: Distribution of scores obtained by searching Hox PG9 profile against different protein databases: Swissprot, Swissprot excluding PG9 sequences, randomized Swissprot and random sequences generated to display the same lengths as Swissprot. The results for the two types of random protein sequences are similar. T : theoretical threshold. S : threshold that discriminate PG9 sequences. These random sequences have very low scores (<820) and present the same distribution of scores as most Swissprot proteins. Several proteins from Swissprot nevertheless show higher scores, and should theorically be significant matches. Actually, sequences having a score between 820 and 5230 correspond to Hox proteins belonging to non-PG9 groups as well as non-Hox homeobox proteins. Excluding the Hox PG9 proteins from Swissprot reveals that only high scoring matches (>5230) correspond to Hox PG9 proteins.
