# Supplementary material for: HoxPred: automated classification of Hox proteins using combinations of generalised profiles
Source: BMC Bioinformatics. 2007 Jul 12;8:247. doi: 10.1186/1471-2105-8-247 (PMC1965487; doi:10.1186/1471-2105-8-247)
Supplement: Additional File 5 — Application of HoxPred to medaka and stickleback homeobox proteins [file 1471-2105-8-247-S5.pdf]

<table>
<tr><td colspan="6">Oryzia latipes</td><td colspan="6">Gasterosteus aculeatus</td></tr>
<tr><td>chr|accession no</td><td>length</td><td>PG</td><td>pp</td><td>OG</td><td>pp</td><td>group|accession no</td><td>length</td><td>PG</td><td>pp</td><td>OG</td><td>pp</td></tr>
<tr><td colspan="12">Aa cluster</td></tr>
<tr><td>11|ENSORLG00000004981</td><td>329</td><td>PG1</td><td>1</td><td>A1</td><td>0.99</td><td>groupX|ENSGACG00000007085</td><td>329</td><td>PG1</td><td>1</td><td>A1</td><td>0.92</td></tr>
<tr><td>11|ENSORLG00000004987</td><td>360</td><td>PG2</td><td>1</td><td>A2</td><td>0.97</td><td>groupX|ENSGACG00000007090</td><td>364</td><td>PG2</td><td>1</td><td>A2</td><td>0.97</td></tr>
<tr><td>11|ENSORLG00000004996</td><td>417</td><td>PG3</td><td>1</td><td>A3</td><td>0.88</td><td>groupX|ENSGACG00000007094</td><td>418</td><td>PG3</td><td>1</td><td>A3</td><td>0.88</td></tr>
<tr><td>11|ENSORLG00000005025</td><td>764</td><td>PG4</td><td>1</td><td>A4</td><td>1</td><td>groupX|ENSGACG00000007100</td><td>251</td><td>PG4</td><td>1</td><td>A4</td><td>1</td></tr>
<tr><td>11|ENSORLG00000005025</td><td>186</td><td>PG5</td><td>1</td><td>A5</td><td>1</td><td>groupX|ENSGACG00000007108</td><td>278</td><td>PG5</td><td>1</td><td>A5</td><td>1</td></tr>
<tr><td></td><td></td><td></td><td></td><td></td><td></td><td>groupX|ENSGACG00000007112</td><td>226</td><td>PG7</td><td>1</td><td>A7</td><td>1</td></tr>
<tr><td>11|ENSORLG00000005025</td><td>270</td><td>PG9</td><td>1</td><td>A9</td><td>1</td><td>groupX|ENSGACG00000007123</td><td>241</td><td>PG9</td><td>1</td><td>A9</td><td>1</td></tr>
<tr><td>11|ENSORLG00000005034</td><td>363</td><td>PG10</td><td>1</td><td>A10</td><td>1</td><td>groupX|ENSGACG00000007128</td><td>308</td><td>PG10</td><td>1</td><td>A10</td><td>1</td></tr>
<tr><td>11|ENSORLG00000005037</td><td>300</td><td>PG11</td><td>1</td><td>A11</td><td>1</td><td>groupX|ENSGACG00000007132</td><td>291</td><td>PG11</td><td>1</td><td>A11</td><td>1</td></tr>
<tr><td>11|ENSORLG00000005043</td><td>300</td><td>PG13</td><td>1</td><td>A13</td><td>1</td><td>groupX|ENSGACG00000007134</td><td>304</td><td>PG13</td><td>1</td><td>A13</td><td>1</td></tr>
<tr><td colspan="12">Ab cluster</td></tr>
<tr><td>16|ENSORLG00000007624</td><td>357</td><td>PG2</td><td>1</td><td>A2</td><td>1</td><td>groupXX|ENSGACG00000008314</td><td>348</td><td>PG2</td><td>1</td><td>A2</td><td>1</td></tr>
<tr><td>16|ENSORLG00000007620</td><td>248</td><td>PG9</td><td>1</td><td>A9</td><td>1</td><td>groupXX|ENSGACG00000008310</td><td>254</td><td>PG9</td><td>1</td><td>A9</td><td>1</td></tr>
<tr><td>16|ENSORLG00000007617</td><td>321</td><td>PG10</td><td>1</td><td>A10</td><td>1</td><td>groupXX|ENSGACG00000008303</td><td>330</td><td>PG10</td><td>1</td><td>A10</td><td>1</td></tr>
<tr><td>16|ENSORLG00000007608</td><td>273</td><td>PG11</td><td>1</td><td>A11</td><td>1</td><td>groupXX|ENSGACG00000008297</td><td>280</td><td>PG11</td><td>1</td><td>A11</td><td>1</td></tr>
<tr><td>16|ENSORLG00000007597</td><td>298</td><td>PG13</td><td>1</td><td>A13</td><td>1</td><td>groupXX|ENSGACG00000008296</td><td>291</td><td>PG13</td><td>1</td><td>A13</td><td>1</td></tr>
<tr><td colspan="12">Ba cluster</td></tr>
<tr><td>8|ENSORLG00000017053</td><td>395</td><td>PG1</td><td>1</td><td>B1</td><td>0.99</td><td>groupXI|ENSGACG00000005635</td><td>389</td><td>PG1</td><td>1</td><td>B1</td><td>1</td></tr>
<tr><td>8|ENSORLG00000017051</td><td>417</td><td>PG2</td><td>1</td><td>A2</td><td>0.97</td><td>groupXI|ENSGACG00000005633</td><td>438</td><td>PG2</td><td>1</td><td>A2</td><td>0.97</td></tr>
<tr><td>8|ENSORLG00000017048</td><td>288</td><td>PG3</td><td>1</td><td>B3</td><td>0.77</td><td>groupXI|ENSGACG00000005631</td><td>459</td><td>PG3</td><td>1</td><td>B3</td><td>0.77</td></tr>
<tr><td>8|ENSORLG00000017046</td><td>270</td><td>PG4</td><td>1</td><td>B4</td><td>1</td><td>groupXI|ENSGACG00000005628</td><td>258</td><td>PG4</td><td>1</td><td>B4</td><td>1</td></tr>
<tr><td>8|ENSORLG00000017041</td><td>312</td><td>PG5</td><td>1</td><td>B5</td><td>1</td><td>groupXI|ENSGACG00000005626</td><td>310</td><td>PG5</td><td>1</td><td>B5</td><td>1</td></tr>
<tr><td>8|ENSORLG00000017036</td><td>210</td><td>PG6</td><td>1</td><td>B6</td><td>1</td><td>groupXI|ENSGACG00000005623</td><td>94</td><td>PG6</td><td>1</td><td>B6</td><td>1</td></tr>
<tr><td>8|ENSORLG00000017035</td><td>283</td><td>PG9</td><td>1</td><td>B9</td><td>1</td><td></td><td></td><td></td><td></td><td></td><td></td></tr>
<tr><td>8|ENSORLG00000017031</td><td>327</td><td>PG13</td><td>1</td><td>B13</td><td>1</td><td>groupXI|ENSGACG00000005617</td><td>321</td><td>PG13</td><td>1</td><td>B13</td><td>1</td></tr>
<tr><td colspan="12">Bb cluster</td></tr>
<tr><td>19|ENSORLG00000012369</td><td>279</td><td>PG1</td><td>1</td><td>B1</td><td>0.90</td><td>groupV|ENSGACG00000003939</td><td>264</td><td>PG1</td><td>1</td><td>B1</td><td>0.99</td></tr>
<tr><td>19|ENSORLG00000012375</td><td>263</td><td>PG3</td><td>1</td><td>B3</td><td>0.99</td><td>groupV|ENSGACG00000003942</td><td>190</td><td>PG3</td><td>1</td><td>B3</td><td>1</td></tr>
<tr><td>19|ENSORLG00000012379</td><td>282</td><td>PG5</td><td>1</td><td>B5</td><td>1</td><td>groupV|ENSGACG00000003945</td><td>246</td><td>PG5</td><td>1</td><td>B5</td><td>1</td></tr>
<tr><td>19|ENSORLG00000012387</td><td>236</td><td>PG6</td><td>1</td><td>B6</td><td>1</td><td></td><td></td><td></td><td></td><td></td><td></td></tr>
<tr><td colspan="12">Ca cluster</td></tr>
<tr><td></td><td></td><td></td><td></td><td></td><td></td><td>groupXII|ENSGACG00000009431</td><td>107</td><td>PG1</td><td>1</td><td>B1</td><td>1</td></tr>
<tr><td>7|ENSORLG00000007924</td><td>236</td><td>PG3</td><td>1</td><td>C3</td><td>1</td><td>groupXII|ENSGACG00000009430</td><td>177</td><td>PG3</td><td>1</td><td>C3</td><td>1</td></tr>
<tr><td>7|ENSORLG00000007955</td><td>264</td><td>PG4</td><td>1</td><td>C4</td><td>1</td><td>groupXII|ENSGACG00000009421</td><td>265</td><td>PG4</td><td>1</td><td>C4</td><td>1</td></tr>
<tr><td>7|ENSORLG00000007942</td><td>229</td><td>PG5</td><td>1</td><td>C5</td><td>1</td><td>groupXII|ENSGACG00000009416</td><td>230</td><td>PG5</td><td>1</td><td>C5</td><td>1</td></tr>
<tr><td></td><td></td><td></td><td></td><td></td><td></td><td>groupXII|ENSGACG00000009405</td><td>236</td><td>PG6</td><td>1</td><td>C6</td><td>1</td></tr>
<tr><td>7|ENSORLG00000007935</td><td>251</td><td>PG8</td><td>1</td><td>C8</td><td>1</td><td>groupXII|ENSGACG00000009401</td><td>310</td><td>PG8</td><td>1</td><td>C8</td><td>1</td></tr>
<tr><td>7|ENSORLG00000007935</td><td>270</td><td>PG9</td><td>1</td><td>C9</td><td>0.78</td><td>groupXII|ENSGACG00000009396</td><td>263</td><td>PG9</td><td>1</td><td>C9</td><td>0.93</td></tr>
<tr><td>7|ENSORLG00000007913</td><td>327</td><td>PG10</td><td>1</td><td>C10</td><td>1</td><td>groupXII|ENSGACG00000009394</td><td>327</td><td>PG10</td><td>1</td><td>C10</td><td>1</td></tr>
<tr><td>7|ENSORLG00000007909</td><td>315</td><td>PG11</td><td>1</td><td>D11</td><td>1</td><td>groupXII|ENSGACG00000009392</td><td>310</td><td>PG11</td><td>1</td><td>D11</td><td>1</td></tr>
<tr><td>7|ENSORLG00000007902</td><td>273</td><td>PG12</td><td>1</td><td>C12</td><td>1</td><td>groupXII|ENSGACG00000009391</td><td>268</td><td>PG12</td><td>1</td><td>C12</td><td>1</td></tr>
<tr><td>7|ENSORLG00000007896</td><td>307</td><td>PG13</td><td>1</td><td>C13</td><td>1</td><td>groupXII|ENSGACG00000009389</td><td>307</td><td>PG13</td><td>1</td><td>C13</td><td>1</td></tr>
<tr><td colspan="12">Da cluster</td></tr>
<tr><td>21|ENSORLG00000017546</td><td>400</td><td>PG3</td><td>1</td><td>D3</td><td>0.94</td><td>groupXVI|ENSGACG00000004548</td><td>395</td><td>PG3</td><td>1</td><td>D3</td><td>0.94</td></tr>
<tr><td>21|ENSORLG00000017548</td><td>237</td><td>PG4</td><td>1</td><td>D4</td><td>1</td><td>groupXVI|ENSGACG00000004551</td><td>235</td><td>PG4</td><td>1</td><td>D4</td><td>1</td></tr>
<tr><td>21|ENSORLG00000017544</td><td>274</td><td>PG9</td><td>1</td><td>D9</td><td>0.70</td><td>groupXVI|ENSGACG00000004556</td><td>164</td><td>PG9</td><td>1</td><td>D9</td><td>0.90</td></tr>
<tr><td>21|ENSORLG00000017540</td><td>351</td><td>PG10</td><td>1</td><td>D10</td><td>1</td><td>groupXVI|ENSGACG00000004564</td><td>338</td><td>PG10</td><td>1</td><td>D10</td><td>0.99</td></tr>
<tr><td>21|ENSORLG00000017538</td><td>275</td><td>PG11</td><td>1</td><td>D11</td><td>1</td><td>groupXVI|ENSGACG00000004569</td><td>305</td><td>PG11</td><td>1</td><td>D11</td><td>1</td></tr>
<tr><td>21|ENSORLG00000017536</td><td>265</td><td>PG12</td><td>1</td><td>D12</td><td>1</td><td>groupXVI|ENSGACG00000004574</td><td>265</td><td>PG12</td><td>1</td><td>D12</td><td>1</td></tr>
<tr><td colspan="12">Db cluster</td></tr>
<tr><td>15|ENSORLG00000001346</td><td>255</td><td>PG4</td><td>1</td><td>D4</td><td>1</td><td>groupVI|ENSGACG00000011907</td><td>247</td><td>PG4</td><td>1</td><td>D4</td><td>1</td></tr>
<tr><td>15|ENSORLG00000001349</td><td>143</td><td>PG9</td><td>1</td><td>D9</td><td>1</td><td>groupVI|ENSGACG00000011902</td><td>281</td><td>PG9</td><td>1</td><td>D9</td><td>1</td></tr>
<tr><td colspan="6">not arranged on a cluster</td><td>groupXVII|ENSGACG00000003421</td><td>321</td><td>PG13</td><td>1</td><td>B13</td><td>1</td></tr>
<tr><td colspan="6">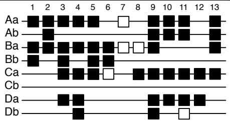</td><td colspan="6">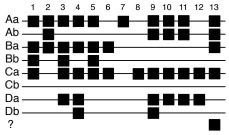</td></tr>
</table>

pp: posterior probability. The sketch depicts Hox genes organisation for medaka and stickleback, deduced from HoxPred predictions and positions in the genome assembly. Symbols: black square, identified gene ; white square, hypothetical gene reported in Hoegg, 2005, not present in Ensembl.
