# Supplementary material for: HoxPred: automated classification of Hox proteins using combinations of generalised profiles
Source: BMC Bioinformatics. 2007 Jul 12;8:247. doi: 10.1186/1471-2105-8-247 (PMC1965487; doi:10.1186/1471-2105-8-247)
Supplement: Additional File 6 — Phylogenetic tree of PG1 in selected teleost fishes [file 1471-2105-8-247-S6.pdf]

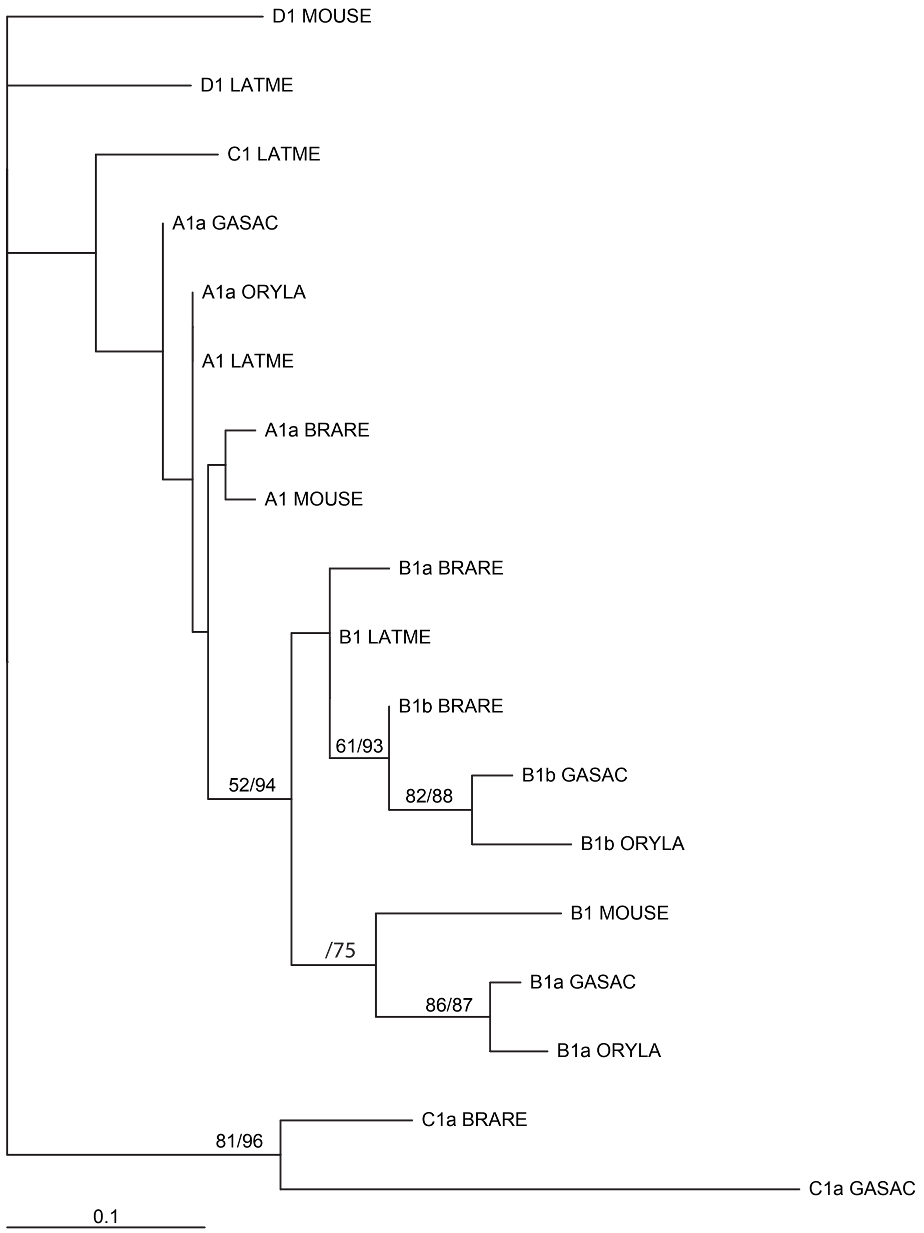
D1 MOUSE
D1 LATME
C1 LATME
A1a GASAC
A1a ORYLA
A1 LATME
A1a BRARE
A1 MOUSE
B1a BRARE
B1 LATME
B1b BRARE
61/93
B1b GASAC
82/88
52/94
B1b ORYLA
B1 MOUSE
/75
B1a GASAC
86/87
B1a ORYLA
C1a BRARE
81/96
C1a GASAC
0.1



Figure 1:

**Phylogenetic tree of the Paralogous group (PG) 1**.

The represented tree is a maximum-likelihood (ML) tree produced by PHYML (Guindon and Gascuel, 2003). Rooting is arbitrary. The first numbers above the internal branches are their bootstrap values. The second numbers correspond to posterior probabilities obtained by Bayesian inference (BI). Only statistical support values > 70 for at least one of the methods used (ML or BI) are shown. Multiple alignments were performed with Clustal W (Thompson et al., 1994). The amino-acid substitution model, Jones-Taylor-Thornton (JTT) (Jones et al., 1992), was choosen using ModelGenerator (Keane et al., 2006). Maximum likelihood (ML) analyses were performed with PHYML using the JTT model, the frequencies of amino acids being estimated from the data set, and rate heterogeneity across sites being modelled by two rate categories (one constant and eight gamma-rates). Statistical support for the different internal branches was assessed by bootstrap resampling (100 bootstrap replicates), as implemented in PHYML. Baysian inference was performed using the Markov chain Monte Carlo method as implemented in the MRBAYES package (version 3) (Huelsenbeck and Ronquist, 2001; Ronquist and Huelsenbeck, 2003), using the JTT model, with among-sites rate variation modelled by means of a discrete gamma distribution with four equally probable categories. Two independent Markov chains were run, each containing from 1,000,000 Monte Carlo. One out of every 200 trees was saved. The trees obtained in the two runs were meshed and the first 25% of the trees were discarded as burnin . Majority consensus of the obtained trees were computed by means of the PAUP 4.0 program. Marginal probabilities at each internal branches were taken as a measure of statistical support. All the alignements and the trees are available upon request.

**References**

Guindon S, Gascuel O: A simple, fast, and accurate algorithm to estimate large phylogenies by maximum likelihood. Syst Biol 2003, 52:696-704.

Huelsenbeck JP, Ronquist F: MRBAYES : Bayesian inference of phylogenetic trees. Bioinformatics 2001, 17:754-755.

Hoegg, S. and Meyer, A. (2005). Hox clusters as models for vertebrate genome evolution. Trends Genet, 21(8), 421424.

Jones DT, Taylor WR, Thornton JM: The rapid generation of mutation data matrices from protein sequences. Comput Appl Biosci 1992, 8:275282.

Keane TM, Creevey CJ, Pentony MM, Naughton TJ, Mclnerney JO. Assessment of methods for amino acid matrix selection and their use on empirical data shows that ad hoc assumptions for choice of matrix are not justified. BMC Evol Biol. 2006, 6: 29.

Ronquist F, Huelsenbeck JP : MrBayes 3: Bayesian phylogenetic inference under mixed models. Bioinformatics 2003, 19:1572-1574.

Thompson JD, Higgins JD, Gibson TJ: CLUSTALW: improving the sensitivity of progressive multiple sequence alignment through sequence weighting, position-specific gap penalties and weight matrix choice. Nucleic Acids Res 1994, 22:46734680.
